# HEARING IMPAIRMENT, DEPRESSION, AND THE ROLE OF SOCIAL ACTIVITY AMONG CHINESE OLDER ADULTS

**DOI:** 10.1093/geroni/igz038.595

**Published:** 2019-11-08

**Authors:** Shu Xu, Haowei Wang, Caitlin Connelly

**Affiliations:** University of Massachusetts Boston, Boston, Massachusetts, United States

## Abstract

Studies suggest that depression is closely linked to hearing impairment, which is highly prevalent among older adults in the United States. There is evidence that social engagement may be impacted by hearing impairment in older adults. However, there is relatively little research on these associations among Chinese older adults. This study examines the relationships between hearing impairment, social activities, and depressive symptoms among older adults in China. Using nationally representative data from the China Health and Retirement Longitudinal Study 2011, we conducted cross-sectional analysis on adults age 60 years and older (n=10,994). Depressive symptoms were assessed by the 10-item Center for Epidemiologic Studies Depression scale and we considered self-reported hearing status (if participants wear a hearing aid and how they would rate their hearing), and social activities (i.e., volunteering, dancing, attending courses, etc.). Models were controlled for age, gender, education, and other covariates. Descriptive analysis showed that 9% of older adults experienced hearing impairment. Multiple linear regression analyses revealed that hearing impairment was positively associated with depressive symptoms among older Chinese adults (β=1.32, p<.001). Social activities were found to partially mediate the relationship between hearing status and depressive symptoms. Respondents with hearing impairment were less likely to engage in social activities (OR=.78, p<.01) and those who did not participate in social activities reported more depressive symptoms (β=1.28, p<.001). These findings suggest that Chinese older adults experiencing hearing loss are at greater risk of depression and that social activities play an important role in the relationship between hearing status and depression.

